# Theoretical Model of EphA2-Ephrin A1 Inhibition

**DOI:** 10.3390/molecules23071688

**Published:** 2018-07-11

**Authors:** Wiktoria Jedwabny, Alessio Lodola, Edyta Dyguda-Kazimierowicz

**Affiliations:** 1 Department of Chemistry, Wrocław University of Science and Technology, 50370 Wrocław, Poland; wiktoria.jedwabny@pwr.edu.pl; 2 Department of Food and Drug, University of Parma, 43100 Parma, Italy; alessio.lodola@unipr.it

**Keywords:** EphA2-ephrin A1, PPI inhibition, interaction energy

## Abstract

This work aims at the theoretical description of EphA2-ephrin A1 inhibition by small molecules. Recently proposed ab initio-based scoring models, comprising long-range components of interaction energy, is tested on lithocholic acid class inhibitors of this protein–protein interaction (PPI) against common empirical descriptors. We show that, although limited to compounds with similar solvation energy, the ab initio model is able to rank the set of selected inhibitors more effectively than empirical scoring functions, aiding the design of novel compounds.

## 1. Introduction

The erythropoietin-producing hepatocellular carcinoma (Eph) receptors are probably the largest family of receptor tyrosine kinases (RTKs) and includes 14 members [1] divided into class A (EphA) and class B (EphB), based on the binding affinity for their ligands (ephrins, also divided into classes A and B), and sequence homology [2]. Ephrins are membrane proteins with the A class connected to the membrane by a phosphatidylinositol (GPI) linker, and the B class linked via a hydrophobic domain. While interclass binding has been reported [3,4], ephrin A-type ligands generally bind to EphA receptors, whereas ephrin B-type ligands interact with EphB receptors.

The Eph-ephrin signaling system is known to play important and diverse biological functions that involve cell–cell interactions both during embryonic development and for maintaining homeostasis in adult cells. For instance, in embryos, the Eph-ephrin system finely tunes tissue boundary formation, including central nervous system patterning [5], while in adults it controls bone and intestinal homeostasis, immune system functions and angiogenic processes. The Eph-ephrin system is currently gaining interest in the context of drug discovery as it has been found hyperactivated in several cancers [6]. Among the cloned Eph receptor subtype, EphA2 has been studied the most in the oncology field since the overexpression and/or the hyperactivation of this receptor has been linked to the insurgence and progression of several cancer types, including brain, lung, breast, ovarian and prostate [7]. Moreover, the abnormal activity of this receptor has been associated with poor prognosis [8]. Due to its increasing recognition as a tumorigenic protein, the EphA2 receptor has gained interest as a target protein for novel cancer therapies [9].

One of the available approaches targeting Eph-ephrin system (and EphA2 with its physiological ligand, ephrin-A1, in particular) involves small molecule inhibitors [1] able to prevent ephrin-A1 binding to EphA2. Several classes of inhibitors of this specific protein–protein interaction (PPI) have been recently identified [10,11,12]. The most promising class is represented by lithocholic acid (LCA) and its α-amino acid conjugates [7,13]. It has been demonstrated by surface plasmon resonance (SPR) analysis that this class of compounds prevents ephrin-A1 binding to EphA2 by targeting a conserved region of the ligand-binding domain of EphA2 [14,15].

Molecular modeling investigations performed with classical force fields have identified a likely binding mode for these inhibitors consistent with available structure–activity relationship (SAR) data, i.e., proposing a reasonable role for the terminal carboxylic group and the amino acid side-chain of the inhibitors during their docking within EphA2 [13,14]. However, attempts to build quantitative models correlating experimental activities to docking energies led to modest results [13], suggesting that classical methods may not be able to properly describe accommodation of amino acid conjugates of LCA within EphA2 ligand binding domain (LBD).

Ligand-receptor binding is often examined using empirical or semi-empirical methods with a diverse level of success [16,17,18,19], particularly in terms of the virtual screening campaigns. A way to improve the quality of the results could involve ab initio calculations, but due to the computational time required, these are rather impractical in the screening of potential drug candidates. On the other hand, quantum chemical calculations are able to provide insight into the physical nature of the receptor–ligand interactions. Studying small-molecule PPI inhibition is usually more challenging than evaluation of interactions in regular protein–ligand complexes [20]. For instance, binding cavities for inhibitors targeting PPIs are flat and often featured by the presence of aromatic residues, such as Phe, Tyr or Trp residues [21]. Empirical scoring functions, commonly used for scoring of receptor–ligand interactions, are not really suited for PPIs [22,23]. Despite the fact that some empirical and semi-empirical approaches have been applied to score PPI inhibitors with moderate success [24,25,26,27], ab initio derived models appear to be better suited for studying PPI recognition by small molecules, since they offer a detailed insight into the physical basis of such interactions.

When polar or charged systems are investigated, the computationally inexpensive non-empirical electrostatic term is sufficient to model the experimental data [28,29]. However, accounting for the dispersive interactions is required for a general description targeting any receptor–ligand complex, irrespectively of the physical nature of binding within such a system [30]. While non-empirical evaluation of the multipole electrostatic term conveniently scales with the size of the complex under study as the squared number of atoms, ab initio calculations of dispersion energy are computationally demanding, scaling with at least the fifth power of the number of atomic orbitals. However, dispersion interactions could be approximated, for instance, by the $E_{Das}$ function, which successfully describes non-covalent interactions with atom–atom potentials fitted to reproduce the results of high-level quantum chemical calculations [31,32]. Recently developed non-empirical model comprising long-range terms of interaction energy, i.e., multipole electrostatic moment and dispersion contribution approximated by $E_{Das}$ function [31,32], which offers a great enhancement in the computational time, was already tested on several systems, including essentially non-polar complexes of fatty acid amide hydrolase (FAAH) [33], pteridine reductase 1 (*Tb*PTR1) featuring both dispersive and electrostatic interactions [34], and menin-mixed lineage leukemia (MLL) system [35], in which electrostatic interactions are dominant.

Such an approach neglects, among other entropic contributions, the influence of solvation effects. To include the latter, one would need a much more time-consuming method, for instance free-energy perturbation (FEP), Molecular Mechanics/Poisson-Boltzmann, Molecular Mechanics/Generalized Born Surface Area (MM/GBSA and MM/PBSA, respectively) [36] or Fragment Molecular Orbital (FMO) approach [37]. The quantum chemical methods (like DFT or MP2) are rather not combined with empirical solvation or ligand entropy estimates [36], and therefore they should work only if the neglected contributions to the energy of binding are similar within the studied set of complexes.

In the work presented herein, we attempt to reproduce the experimental ranking of a congeneric series of EphA2-ephrin A1 inhibitors [38] (shown in Table 1) with a recently developed simple ab initio model comprising multipole electrostatic and dispersion contributions, $E_{EL,MTP}^{\left(10\right)}+E_{Das}$. Such a model was previously validated on another set of protein–protein inhibitors [35], and not only the inhibitory activity ranking was reproduced, but novel inhibitors (i.e., not present in the training set) were successfully scored. We show here that if we limit our analysis to a set of EphA2-ephrin A1 inhibitors featuring similar solvation energy, ab initio modeling of the interactions provides computational results which parallel experimental potency data well. Moreover, such a model is able to outperform several commonly used empirical scoring functions.

## 2. Results and Discussion

**Figure 1:**
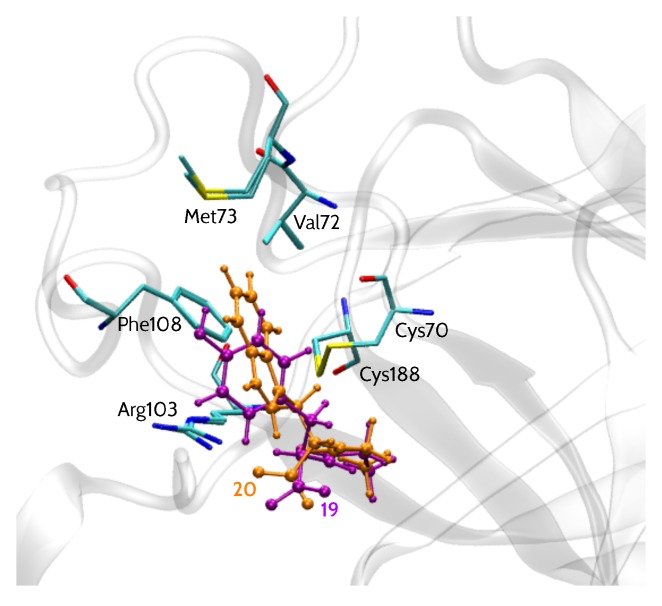
EphA2 binding site representation with bound inhibitors **19** (d-Tyr) and **20** (l-Trp).

EphA2 binding site representation, shown in Figure 1, comprises six amino acid residues: Cys70, Cys188, Phe108, Arg103, Val72 and Met73 (more details regarding the model are given in the Materials and Methods section). All 15 analyzed inhibitors (Table 1) shared a similar binding mode [13], with a –COOH group facing Arg103 residue, in agreement with SAR data. Moreover, their common LCA scaffold was positioned almost identically. Thus, this steroidal moiety was excluded from the analysis and the compounds were cut in a way indicated by the red line in Table 1. Accordingly, the inhibitors were represented by smaller entities corresponding to the variable part of the inhibitor structure. Binding poses of models of two inhibitors, **20** (l-Trp derivative) and **19** (d-Tyr derivative), i.e., the most and least potent compounds, respectively, are presented in Figure 1.

### 2.1. Theoretical Models

Total binding energy values of EphA2 inhibitors for consecutive levels of Hybrid Variation–Perturbation Theory (HVPT) [39,40] and, in addition, $E_{EL,MTP}^{\left(10\right)}+E_{Das}$ energy results, are provided in Table 2. Pairwise interaction energy values between each inhibitor and a given amino acid residue are given in Table S1 in Supplementary Materials. Apparently, the main contribution to the total interaction energy calculated at the MP2 level of theory is due to the electrostatic energy. As a result, $E_{EL}^{\left(10\right)}$ and $E_{MP2}$ energy values are comparable in magnitude (Table 2).

**Figure 2:**
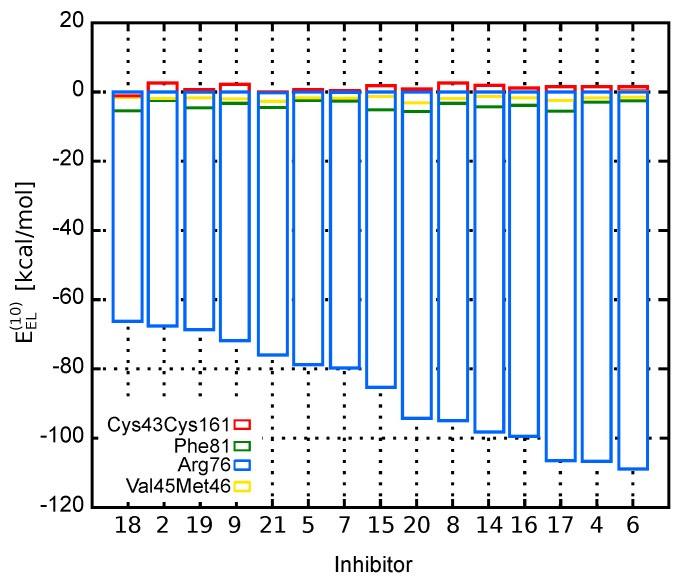
Contribution of EphA2 amino acid residues to the EphA2-inhibitor binding energy represented by the electrostatic term, $E_{EL}^{\left(10\right)}$.

The dominant electrostatic effects appear to arise from the interaction between counter-charged inhibitors and Arg103 residue (charges of −1 and +1, respectively). Indeed, as shown in Figure 2, which presents the electrostatic contribution to the binding energy of each amino acid residue, Arg103–inhibitor interaction has the major impact on the total $E_{EL}^{\left(10\right)}$ energy. Compared to Arg103, the remaining residues are of minor contribution. All inhibitors are directed towards Arg103 residue with their common –COOH group. Thus, any positional inaccuracy of the docked compounds related to Arg103 residue could mask the subtle interactions with other residues.

In general, more potent inhibitors are characterized by higher absolute values of the interaction energy (Table 2). To assess the relationship between the total binding energy and the inhibitory activity, interaction energy terms evaluated within HVPT energy decomposition scheme were correlated with $p{\mathrm{IC}}_{50}$ values established experimentally [13]. It can be seen in Table 2 that the interaction energy results computed at the electrostatic and MP2 levels of theory are comparable in terms of the correlation with the experimental inhibitory activity (R=−0.65 and −0.69, respectively). Correlation coefficient of the multipole electrostatic model of inhibitory activity is slightly lower (R=−0.63), but the values of the statistical predictor $N_{pred}$ (the success rate of prediction of relative affinities, explained further in the Materials and Methods section) are comparable for all three levels of theory and remain within the range between 75.0% ($E_{EL,MTP}^{\left(10\right)}$, $E_{MP2}$) and 76.9% ($E_{EL}^{\left(10\right)}$). The first order Heitler–London energy ($E^{\left(10\right)}$) is characterized by the weakest relationship with the experimental inhibitory activity (R=−0.44, Table 2), which is due to the repulsive $E_{EX}^{\left(10\right)}$ term of the interaction energy. Apparently, the short-range exchange term of the interaction energy has contributed to the greatest extent to the binding of inhibitors with higher affinity to the EphA2 LBD, resulting in the drop of the R value at the $E^{\left(10\right)}$ level of theory. It has already been observed for other complexes [29,34] that structures obtained with empirical docking protocols and further evaluated with ab initio methods appear to suffer from the presence of artificially shortened intermolecular distances. Due to the sensitivity of short-range interaction energy components to any structural deficiencies, long-range binding energy terms seem to be more suitable for the determination of the relative ligand binging affinities [41]. Thus, the following $E_{SCF}$ level of theory, which accounts for short-range delocalization contribution ($E_{DEL}^{\left(R0\right)}$), is only slightly improved compared to $E^{\left(10\right)}$ in terms of the correlation (R=−0.55, Table 2). Nevertheless, only the introduction of the correlation term $E_{CORR}^{\left(2\right)}$, that is present in $E_{MP2}$ energy, is able to recover the predictive abilities of the inhibitory activity model, as the corresponding correlation coefficient amounts to −0.69. Similarly to values of the Pearson correlation coefficient, $N_{pred}$ values associated with $E^{\left(10\right)}$ and $E_{SCF}$ are also lower compared to the statistical outcome obtained for the remaining levels of theory.

Among all presented levels of theory, $E_{EL,MTP}^{\left(10\right)}+E_{Das}$ model offers the best performance (R=−0.72 or $R^{2}=0.52$, $N_{pred}=77.9\%$). Reasonable agreement with experimental binding potency yielded by $E_{EL,MTP}^{\left(10\right)}+E_{Das}$ model indicates that accounting only for long-range interaction energy terms could compete with the computationally expensive MP2 level of theory. Still, its predictive abilities for EphA2-ephrin A1 inhibitors appear to be rather limited. Therefore, the impact of solvation was further analyzed to check whether it might be significant in this particular system.

### 2.2. Solvation Energy of Inhibitors

PPI contact surfaces are large [42], and the targeted EphA2 receptor fits into this description. Therefore, with a small molecule inhibitor bound, the EphA2 binding site remains relatively solvent exposed. As a result, solvation effects could possibly affect the interaction energy and influence the correlation between the latter and the experimental binding affinities. On the other hand, in the case of inhibition of another PPI system, i.e., menin-MLL complex [35], the nonempirical model accounting for the gas phase interaction only was sufficient to reproduce the experimental data. This could arise from the fact that substantially more amino acid residues surround menin ligands than in the case of EphA2 receptor. To determine the importance of solvation effects for binding of EphA2-ephrin A1 inhibitors, solvation free energy was calculated for all compounds analyzed herein.

The solvation free energy, $\Delta G_{solv}$, along with its electrostatic and non-electrostatic contributions ($\Delta G_{solv,el}$ and $\Delta G_{solv,non-el}$, respectively), is given in Table 3 for each EphA2 inhibitor. It can be concluded from the analysis of the correlation coefficient values provided in Table 3 that $\Delta G_{solv}$ energy values do not explicitly correlate with the experimental binding potency. Nonempirical models of inhibitory activity applied herein operate under the assumption that the enthalpic contribution to the binding free energy is responsible for the observed differences in ligand binding affinity. Accordingly, applicability of the interaction energy-based nonempirical approaches is limited to the set of ligands characterized by similar solvation free energy. Considering the suboptimal performance of $E_{EL,MTP}^{\left(10\right)}+E_{Das}$ model in predicting the inhibitory activity of EphA2 ligands (R=−0.72, see Table 2), compared to more significant correlation obtained previously for, e.g., menin-MLL inhibitors (R=−0.87 [35]), the possible influence of the solvation effects was further investigated by calculating ΔG of solvation for FAAH [33], *Tb*PTR1 [34] and menin-MLL [35] inhibitors. In all cases, $\Delta G_{solv}$ is calculated at the MP2 level of theory, but the basis sets used depend on the system (FAAH: 6-31G(d), menin-MLL: 6-31G(d), *Tb*PTR1: 6-311G(d) with diffuse functions on S and P orbitals of chlorine atoms; the choice of basis set was made to match the remaining ab initio interaction energy calculations performed for each of these systems). The solvation free energies of FAAH, *Tb*PTR1 and menin-MLL inhibitors (22, 6, and 18 inhibitors in each system, respectively) are given in Supplementary Materials in Tables S2–S4. Comparison of the corresponding $\Delta G_{solv}$ standard deviation is provided in Table 4 for all abovementioned inhibitors.

Among the ligand sets presented in Table 4, EphA2-ephrin A1 inhibitors are characterized by the largest value of standard deviation of solvation free energy ($3.0\;\mathrm{kcal}\cdot {\mathrm{mol}}^{-1}$). Since the linear relationship between interaction energy and experimental affinities assumes, among other factors, that the solvation effects are comparable for all inhibitors within the set, this could indicate that this expectation is not met in the case of EphA2-ephrin A1 inhibitors. Considering that PCM results can be obtained easily, $\Delta G_{solv}$ standard deviation could be used as an initial predictor of the applicability of $E_{EL,MTP}^{\left(10\right)}+E_{Das}$ model.

**Figure 3:**
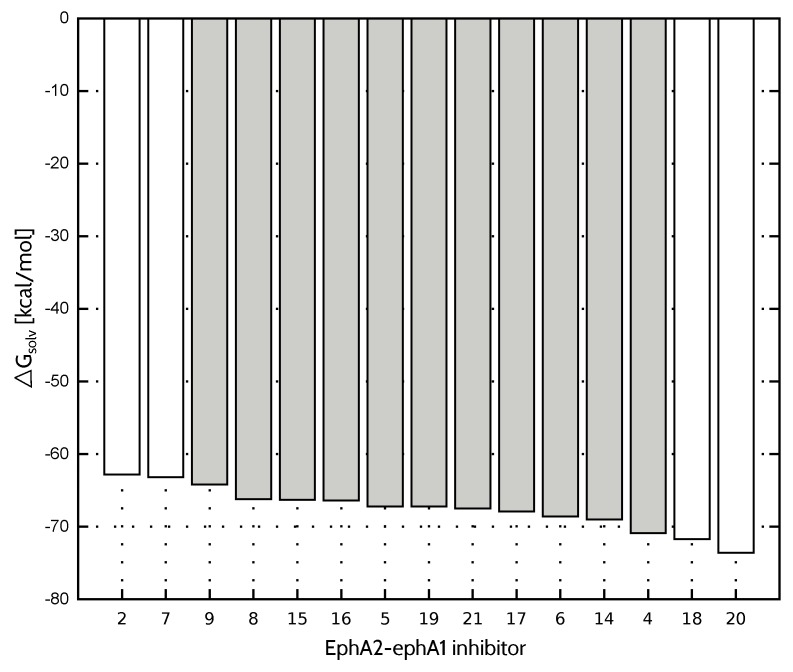
Solvation free energy of EphA2-ephA1 inhibitors. Compounds indicated in white were not included in the reduced ligand set.

**Figure 4:**
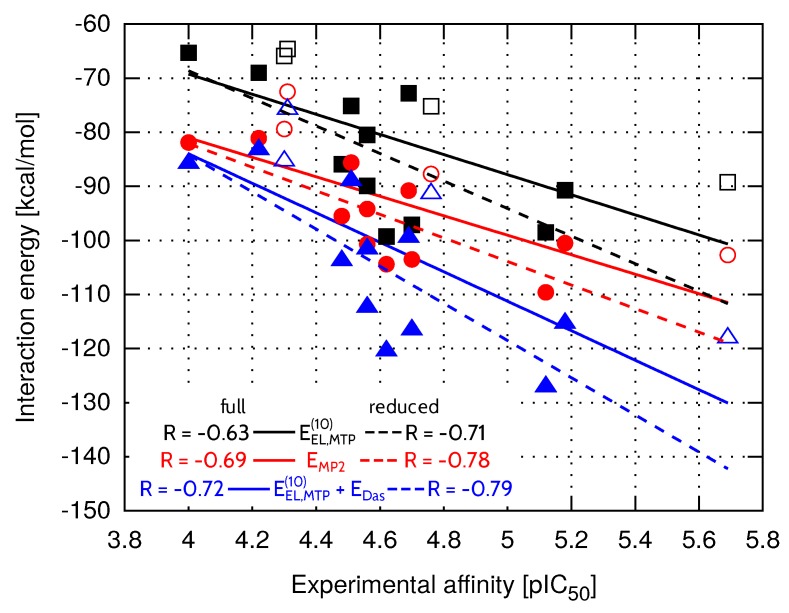
Total EphA2-inhibitor interaction energy at the selected levels of theory within the full (solid line) and reduced (dashed line) ligand sets. The reduced set of EphA2 inhibitors consists of the compounds shown with full symbols.

Compared to FAAH and *Tb*PTR1 ligand sets, characterized by significantly lower values of $\Delta G_{solv}$ standard deviation (Table 4), $E_{MP2}$ model provides less accurate inhibitory activity predictions in the case of both EphA2-ephrin A1 and menin-MLL systems. On the other hand, the best performing $E_{EL,MTP}^{\left(10\right)}+E_{Das}$ model is not able to predict the EphA2-ephrin A1 inhibitory activity to the extent observed for menin-MLL or *Tb*PTR1 inhibitors. Therefore, it seemed interesting if omitting the inhibitors that differ the most in terms of $\Delta G_{solv}$ values (compounds **20**, **7**, **2** and **18**, all marked in white in Figure 3) would improve the results. The standard deviation of solvation free energy associated with the resulting reduced set of EphA2 inhibitors is equal to $1.8\;\mathrm{kcal}\cdot {\mathrm{mol}}^{-1}$. The correlation coefficients obtained for the full and reduced ligand sets are compared in Figure 4 for $E_{EL,MTP}^{\left(10\right)}$, $E_{MP2}$, and $E_{EL,MTP}^{\left(10\right)}+E_{Das}$ models. The corresponding correlation coefficients and $N_{pred}$ values for all the nonempirical models of inhibitory activity, as applied to the full and reduced ligands sets, are provided in the Supplementary Materials (Table S5). Indeed, the reduced set of EphA2 inhibitors, obtained by selecting the compounds with essentially similar solvation free energies (Figure 3) features improved values of correlation coefficients. In particular, $E_{EL,MTP}^{\left(10\right)}+E_{Das}$ model provides the most accurate predictions (Figure 4), as the corresponding correlation coefficient *R* amounts to −0.79 ($R^{2}=0.62$).

Overall, selection of ligands to be excluded based on their $\Delta G_{solv}$ differences is rather an arbitrary approach, as one could iteratively select inhibitors to reach even lower standard deviation values and, presumably, better predictive abilities of the nonempirical approach. On the other hand, a more extensive elimination of compounds does not necessarily improve the correlation coefficient between the given interaction energy model and the experimental binding potency. It can be seen in Figure 3 that ligands **4** and **9** feature $\Delta G_{solv}$ values similar to compounds **2**, **7**, **18** and **20**, already exluded from the initial set due to solvation free energy differing the most in comparison with the majority of EphA2 inhibitors considered herein. However, further limiting the size of the test set by removal of compounds **4** and **9** results in no improvement in the correlation coefficient values (R=−0.75 and −0.76 for $E_{MP2}$ and $E_{EL,MTP}^{\left(10\right)}+E_{Das}$, respectively), despite substantial drop in the $\Delta G_{solv}$ standard deviation equal to $1.0\;\mathrm{kcal}\cdot {\mathrm{mol}}^{-1}$. It should be noted that since the models of receptor–ligand complexes are developed with certain approximations due to the lack of experimental structures, they cannot be expected to provide perfect agreement with the experimental binding potency. Therefore, the ligand elimination based on the $\Delta G_{solv}$ differences also appears to be a limited approach. Nevertheless, it provides a reasonable basis for the exclusion of the $\Delta G_{solv}$ outliers with simultaneous improvement in the performance of nonempirical models applied herein.

### 2.3. Empirical Evaluation of EphA2-Ephrin A1 Inhibitors

**Figure 5:**
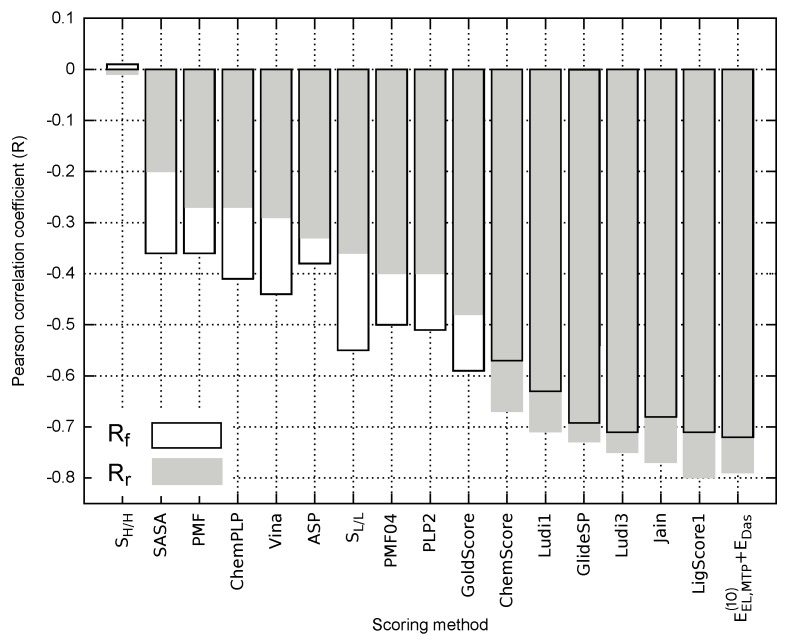
Pearson correlation coefficients obtained for the empirical scoring methods and $E_{EL,MTP}^{\left(10\right)}+E_{Das}$ model applied to the full ($R_{f}$) and reduced ($R_{r}$) ligand sets.

To further evaluate the predictive potential of various empirical descriptors related to receptor–ligand binding, Solvent Accessible Surface Area (SASA) and Molecular Hydrophobicity Potential (MHP) were calculated for each EphA2-ephrin A1 inhibitor. Both lipophilic ($S_{L/L}$) and hydrophilic match surfaces ($S_{H/H}$) obtained with MHP calculation could help to assess the hydrophobic/hydrophilic complementarity of the analyzed ligands to the receptor binding site, which is based on the surface area of favorable (hydrophilic-hydrophilic) and unfavorable (hydrophilic-hydrophobic) contacts [43]. A number of scoring functions were also used for comparison, namely LigScore1 [44], PLP2 [45,46], Jain [47], PMF [48], PMF04 [49], Ludi1 [50], and Ludi3 [51] (available in Discovery Studio 2017 [52]), GoldScore, ChemScore and ASP (implemented in Gold 4.0 program [53]), AutoDock Vina [54], ChemPLP (Plants program [55]), and Glide SP [56]. Correlation coefficients associated with all these empirical approaches are compared in Figure 5 for both full and reduced ligand sets. The numerical data reflecting each empirical score obtained for EphA2 inhibitors alongwith the corresponding correlation coefficients and $N_{pred}$ values are provided in Table S6 in Supplementary Materials.

The best performing empirical descriptors for both full and reduced ligand sets include LigScore1, Jain, Ludi3, GlideSP and Ludi1 (Figure 5). In fact, the related correlation coefficients are comparable with the corresponding value characterizing $E_{EL,MTP}^{\left(10\right)}+E_{Das}$ model, e.g., in the case of full ligand set R=−0.71 ($R^{2}=0.50$) and −0.72 ($R^{2}=0.52$) for LigScore1 and $E_{EL,MTP}^{\left(10\right)}+E_{Das}$, respectively (see Table S6 in Supplementary Materials). Nevertheless, the majority of the analyzed empirical scoring functions yield unsatisfactory results and are outperformed by most of the nonempirical models, including $E_{EL,MTP}^{\left(10\right)}+E_{Das}$. As it has been pointed out by Li et al. [57], SASA appears to perform better as a scoring method than a number of popular scoring functions [57,58]. Accordingly, outperforming the SASA predictions might be viewed as a necessary condition, allowing for distinguishing between the scoring functions providing reasonable results and those failing to reflect the experimental binding affinity. In this particular case, most of the scoring approaches presented in Figure 5 seem to pass this test; however, only some of the empirical approaches, and $E_{EL,MTP}^{\left(10\right)}+E_{Das}$ model in particular, appear to provide at least semi-quantitative agreement with the experimental inhibitory activity.

In contrast to the theoretical models considered herein (Table S5 in Supplementary Materials), the correlation between the empirical scoring functions and experimental inhibitory activity values do not always improve when the reduced model is considered (Figure 5). This could arise from the fact that solvation effects might be implicitly included in the empirical description by parametrization performed with experimental binding potency. Depending on the ability of a given scoring function to account for the influence of solvent, limiting the test ligand set to the inhibitors featuring similar solvation energy might either decrease the performance of the method (PLP2 and PMF04) or improve the predictions, as can be seen for (e.g., LigScore1 and Jain; see Figure 5).

It seemed interesting to check whether there is some consistency in top scoring empirical functions throughout the systems tested so far in our group. Since some scoring functions implemented in Discovery Studio have been used also in the case of FAAH [33] and menin-MLL [35], comparison was made for these methods. The performance of LigScore1, PLP2, Jain, PMF, and Ludi1, described by correlation coefficients and percentage of successful predictions ($N_{pred}$) is presented in Table 5 for FAAH, menin-MLL and EphA2-ephA1 systems. In the latter case, comparison was made based on the results associated with the reduced set of ligands featuring similar $\Delta G_{solv}$ values. As demonstrated in Tables S5 and S6 in Supplementary Materials, selecting EphA2 inhibitors with relatively similar values of solvation free energy improves the performance of both nonempirical $E_{EL,MTP}^{\left(10\right)}+E_{Das}$ model and most of the scoring functions included in this comparison.

It can be seen in Table 5 that both LigScore1 and Jain provide the best prediction for menin-MLL and EphA2-ephrin A1 systems. On the contrary, the performance of these scoring functions is unsatisfactory in the case of FAAH inhibitors. Entirely different predictive abilities seem to be associated with PMF function, that performs the best for FAAH system, yet it fails in the case of both menin and EphA2 inhibitors. As for the remaning empirical scoring functions compared herein, PLP2 appears to provide valid predictions only for menin-MLL system, whereas Ludi1 yields rather satisfactory agreement with the experimental data for both FAAH and EphA2 inhibitors. The interactions in menin-MLL [35] and EphA2-ephrin A1 system are predominantly electrostatic in nature, and it seems that LigScore1 or Jain functions might be better suited in such a case. On the other hand, for dispersion-dominated systems like FAAH [33], PMF could be a better choice. Nevertheless, the performance of $E_{EL,MTP}^{\left(10\right)}+E_{Das}$ model is comparable (or superior, as demonstrated in the case of menin-MLL system) to the best empirical scoring functions in each system analyzed so far. Considering that the physical nature of interactions for novel receptor–ligand complexes can hardly be determined without time-consuming ab initio calculations and the resulting choice of a reliable empirical scoring function might not be clear, the nonempirical $E_{EL,MTP}^{\left(10\right)}+E_{Das}$ model appears to be a preferable method, capable of providing the predictions with a reasonable quality at the computational cost comparable to that of empirical scoring functions.

## 3. Materials and Methods

### 3.1. Preparation of the Structures

From the LCA-based structures reported by Incerti et al. [13], all active α-amino acid LCA conjugates were selected. An LCA compound was not included in this analysis on account of the likely multiple binding modes within EphA2 [14]. In contrast, LCA amino acid conjugates studied herein presumably possess a single binding mode due to the interaction between the carboxylate group and Arg103 residue of EphA2 receptor. The structures of the selected inhibitors and the corresponding $p{\mathrm{IC}}_{50}$ vales (taken from [13]) are given in Table 1.

The geometries of EphA2-inhibitor complexes, obtained from molecular docking simulation [13], were provided by Incerti et al. [13]. Since the goal of the analysis was to investigate the influence of amino acid substituent on the activity of the inhibitors, the common LCA scaffold, positioned similarly in all complexes, was not included in the analysis. In particular, the inhibitors were cut as indicated by the red line in the scaffold representation in Table 1.

To obtain more reliable positions of amino acid residues, all EphA2-inhibitor complexes were solvated with the TIP3 water model [59] and re-optimized in the Charmm program [60] (version c36b1, Harvard University, Cambridge, MA, USA). Hydrogen atoms were built with Hbuild command. Both Charmm General Force Field v. 2b7 [61] and Charmm22 All-Hydrogen Force Field [62,63,64] parameter files were used. Missing parameters for inhibitor structures were generated with CGenFF interface at http://cgenff.paramchem.org [61,65,66,67] (interface version 1.0.0). LCA scaffold and all amino acid residues further than 4 Å from each inhibitor were kept frozen throughout 1000 steps of steepest descent followed by conjugate gradient optimization until a root mean squared deviation of the gradient (GRMS) of $0.01\;\mathrm{kcal}\cdot {\mathrm{mol}}^{-1}\cdot$Å was reached.

The model of EphA2 binding site included all residues in the vicinity of 4 Å of the interchangeable fragment of the inhibitors, i.e., Cys70, Cys188, Phe108, Arg103, Val72 and Met73 (Figure 1). Dangling bonds resulting from cutting the amino acid residues from protein structure were filled with hydrogen atoms minimized in the Schrödinger Maestro [68] program (Maestro version 9.3, Schrödinger, LLC, New York, NY, USA) using Opls 2005 force field [69].

### 3.2. Interaction Energy Calculations

Interaction energy between EphA2 receptor and each inhibitor was calculated within Hybrid Variation–Perturbation Theory (HVPT) [39,40] decomposition scheme as the sum of interaction energy components obtained for each residue-inhibitor dimer. Counterpoise correction was applied in the treatment of the basis set superposition error [70]. The calculations were carried out with a modified version [40] of Gamess program [71] using 6-311+G(d) basis set [72,73,74]. HVPT introduces the partitioning of the Møller–Plesset second-order interaction energy ($E_{MP2}$) into the multipole electrostatic ($E_{EL,MTP}^{\left(10\right)}$), penetration ($E_{EL,PEN}^{\left(10\right)}$), exchange ($E_{EX}^{\left(10\right)}$), delocalization ($E_{DEL}^{\left(R0\right)}$) and correlation ($E_{CORR}^{\left(2\right)}$) terms:

(1) $$\begin{array}{c} E_{MP2}=\overset{R^{-n}}{\overset{︷}{E_{EL,MTP}^{\left(10\right)}}}+\overset{exp\left(-\gamma R\right)}{\overset{︷}{E_{EL,PEN}^{\left(10\right)}+E_{EX}^{\left(10\right)}+E_{DEL}^{\left(R0\right)}}}+\overset{R^{-n}}{\overset{︷}{E_{CORR}^{\left(2\right)}}}\\ O\left(N^{5}\right)\;\underset{E_{MP2}}{\underset{︸}{\;}}\\ O\left(N^{4}\right)\;\underset{E_{SCF}}{\underset{︸}{\;}}\\ O\left(N^{4}\right)\;\underset{E^{\left(10\right)}}{\underset{︸}{\;}}\\ O\left(N^{4}\right)\;\underset{E_{EL}^{\left(10\right)}}{\underset{︸}{\;}}\\ O\left(A^{2}\right)\;\underset{E_{EL,MTP}^{\left(10\right)}}{\underset{︸}{\;}}, \end{array}$$

which could be divided into the long- and short-range contributions that vary with the intermolecular distance *R* as $R^{-n}$ and exp(−γR), respectively. $E_{EL,MTP}^{\left(10\right)}$ term from Equation (1) is the electrostatic multipole component of the binding energy. Herein, it was estimated from Cumulative Atomic Multipole Moment (Camm) expansion (implemented in Gamess), truncated at the $R^{-4}$ term. The first-order electrostatic energy ($E_{EL}^{\left(10\right)}$) is obtained by adding the penetration term, $E_{EL,PEN}^{\left(10\right)}$, to the $E_{EL,MTP}^{\left(10\right)}$ energy. The first-order Heitler–London energy, $E^{\left(10\right)}$, is the sum of first-order electrostatic energy and the exchange component $E_{EX}^{\left(10\right)}$. The higher order delocalization energy, $E_{DEL}^{\left(R0\right)}$, comprising classical induction and charge transfer terms, is defined as the difference between the counterpoise-corrected self-consistent field (SCF) variational energy, $E_{SCF}$, and the first-order Heitler–London energy, $E^{\left(10\right)}$. The correlation term $E_{CORR}^{\left(2\right)}$ is calculated as the difference of the second-order Møller–Plesset interaction energy, $E_{MP2}$, and converged SCF energy, $E_{SCF}$. $E_{CORR}^{\left(2\right)}$ consists mostly of intramolecular correlation contributions, dispersion and exchange-dispersion interaction energy terms. The zero value of the second superscript accompanying some energy terms in Equation (1) represents uncorrelated interaction energy contributions. O(X) in Equation (1) denotes the scaling of the computational cost, where *N* and *A* indicate the number of atomic orbitals and atoms, respectively.

On account of the considerable computational cost of $E_{CORR}^{\left(2\right)}$ term, containing the dispersion contribution, atom–atom potential function $E_{Das}$ [31,32] was calculated to obtain the approximate dispersion energy at a far more affordable computational expense. In contrast to $E_{CORR}^{\left(2\right)}$, computation scaling with at least the fifth power of the number of atomic orbitals, $O(N^{5})$, $E_{Das}$ calculation scales with the square number of atoms, $O(A^{2})$.

Among amino acid residues in the close proximity of a varying fragment of the LCA derivatives, only Arg103 residue is not neutral, bearing +1 charge. Except for Arg103 residue and two polar cysteine residues, linked by disulfide bond, the remaining residues in the model of EphA2 receptor are nonpolar. The negatively charged (−1) ligands could be considered solvent exposed, as their large fragments face water environment. Since Cys70 and Cys188 residues constitute a disulfide bridge, these residues were considered as Cys70-Cys188 dimer interacting with inhibitors. Similarly, the subsequent Val72 and Met73 residues were not separated but treated as Val72-Met73 dimer to interact with all inhibitors. The remaining residues (Arg103 and Phe108) were included separately.

### 3.3. Solvation Energy Calculations

$\Delta G_{solv}$ for each inhibitor was computed at the MP2/6-311+G(d) level of theory in Gaussian09 [75]. The calculations involved Polarizable Continuum Model (PCM) using the integral equation formalism variant (IEFPCM) [76,77,78] and ExternalIteration [79,80], DoVacuum, and SMD [81] options.

### 3.4. Empirical Scoring

Empirical scoring with a variety of methods was performed for EphA2-inhibitor complexes. As scoring in the presence of water molecules appears to have little influence on the quality of predictions [82], solvent molecules were removed from protein–ligand complexes. Solvent Accessible Surface Area (SASA) [83,84] of each inhibitor was calculated in VMD [85,86] (http://www.ks.uiuc.edu/Research/vmd/) with Sasa.tcl script [87] and the sphere radius set to 1.4 Å. Molecular Hydrophobicity Potential (MHP) was calculated in the Platinum program (version 1.0, Laboratory of Biomolecular Modeling, Russian Academy of Sciences, Moscow, Russia) [43]. GoldScore, ChemScore, and Astex Statistical Potential (ASP) were obtained using Gold 4.0 (The Cambridge Crystallographic Data Centre, Cambridge, United Kingdom) [53] with a spherical grid centered at the alpha carbon of Arg103, comprising amino acid residues within 10 Å radius from the point of origin. Plants [55] docking program with its ChemPLP scoring function was employed with a 10 Å radius sphere. PyMOL [88] and PyMOL AutoDock/Vina plugin [89] were used for preparation of the receptor and inhibitors for scoring in AutoDock Vina (version 1.1.2, Molecular Graphics Lab at The Scripps Research Institute, La Jolla, CA, USA). The latter was carried out with 22.5 Å cubic grid. Glide SP [56] (standard precision), implemented in Schrödinger Glide [90], was applied with a 15 Å grid centered on the ligand. The following scoring functions implemented in Discovery Studio 2017 [52] were used: LigScore1 [44], Piecewise Linear Potential, PLP2 [45,46], Jain [47], Potential of Mean Force, PMF [48] and PMF04 [49], Ludi1 [50] and Ludi3 [51]. In all cases, the scoring performed with Discovery Studio 2017 (Dassault Systèmes BIOVIA, San Diego, CA, USA) suite was carried out with a 10 Å radius sphere centered on the ligand. Calculations performed with AutoDock Vina, Plants, Gold, Glide, and Discovery Studio 2017 involved only scoring of the available compounds’ poses to avoid their re-docking, as this would affect the results. While using all these docking programs, the full protein structures were employed. In each case, standard settings were employed, as further described in Supplementary Materials.

### 3.5. Evaluation of the Results

To assess the performance of each scoring model, the Pearson correlation coefficients were calculated with respect to the experimentally determined inhibitory activity values [13]. The scoring functions with higher score indicating the greater binding potency were assigned the opposite values of the calculated correlation coefficient to facilitate the comparison with the non-empirical interaction energy results, assigning lower binding energy values to more potent inhibitors. Another performance measure applied herein involved the statistical predictor $N_{pred}$, constituting the success rate of prediction of relative affinities, and defined as the percentage of concordant pairs with relative stability of the same sign as in the reference experimentally measured activities, evaluated among all pairs of inhibitors [91]. Here, a special case has occurred as two of the examined inhibitors were reported with an identical experimental affinity ($p{\mathrm{IC}}_{50}$ =4.56 for compounds **14** (l-Met) and **15** (d-Met) [13]). This particular pair of inhibitors was not taken into account while evaluating $N_{pred}$ values.

## 4. Conclusions

The binding of inhibitors of EphA2-ephrin A1 system appears to be dominated by electrostatic interactions. Interaction due to the positively charged Arg103 residue constitutes the major contribution to the interaction energy between the receptor and the negatively charged inhibitors. Nevertheless, accounting for dispersion improves the predictive abilities of the theoretical models applied herein. Among the proposed nonempirical approaches characterizing EphA2-ephrin A1 inhibition, $E_{EL,MTP}^{\left(10\right)}+E_{Das}$ model, comprising solely long-range multipole electrostatic and approximate dispersion interactions, appears to be the best performing (R=−0.72, $N_{pred}=77.9\%$) in terms of the agreement with the experimental data.

Furthermore, solvation effects are probably significant in the case of binding of the presented class of EphA2 inhibitors. Rather limited predictive abilities of $E_{EL,MTP}^{\left(10\right)}+E_{Das}$ model could be related to a relatively large standard deviation of solvation free energy of EphA2-ephrin A1 inhibitors. Compared to $\Delta G_{solv}$ standard deviation obtained for ligands in other systems previously studied in our group, this value is higher and thus could indicate the limited applicability of $E_{EL,MTP}^{\left(10\right)}+E_{Das}$ model for this particular case. In fact, once the set of EphA2 inhibitors is restricted to the ligands featuring essentially similar solvation free energy (i.e., without the compounds **2**, **7**, **18**, **20**), the correlation of the theoretical models with the experimental results is improved, with the performance of $E_{EL,MTP}^{\left(10\right)}+E_{Das}$ model characterized by R=−0.79 and $N_{pred}=79.6\%$.

Despite the limitations discussed above, $E_{EL,MTP}^{\left(10\right)}+E_{Das}$ model is able to outperform essentially all of the empirical descriptors tested herein, including the scoring functions implemented in popular docking programs, such as Gold, AutoDock Vina or Plants. Among the empirical approaches tested herein for EphA2 inhibitors, the only scoring functions that perform comparably to $E_{EL,MTP}^{\left(10\right)}+E_{Das}$ model in this particular case involve LigScore1, Jain and Ludi. However, the scoring performance of these functions is hardly general, as it was not satisfactory in some of the systems studied in our group [33,35]. Based on the comparison encompassing FAAH [33], menin-MLL [35] and EphA2-ephrin A1 cases, it could be tentatively stated that LigScore1 or Jain functions might be better suited for systems with predominant electrostatic interactions (e.g., menin-MLL and EphA2-ephrin A1). In contrast, PMF is probably more appropriate for dispersion-dominated systems (FAAH). Irrespectively of the physical nature of the receptor–ligand binding, the nonempirical $E_{EL,MTP}^{\left(10\right)}+E_{Das}$ model yields the inhibitory activity predictions comparable or outperforming the best empirical scoring function in each of these cases, at similar computational cost. While more tests are required to validate the usefulness and general applicability of $E_{EL,MTP}^{\left(10\right)}+E_{Das}$ model, it appears to constitute an advantageous alternative to commonly used empirical scoring approaches.

## Figures and Tables

**Table 1:** The structures and experimental activity ^*a*^ of inhibitors targeting EphA2-ephrin A1 interaction. The numbering of the structures is consistent with Table 1 from [13].

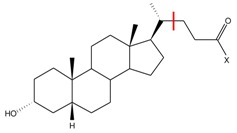		
**Inhibitor**	**X Substituent**	***p*** **IC** ${}_{50}$
**2** (Gly)	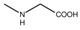	4.31
**4** (l-Ala)	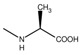	4.70
**5** (d-Ala)	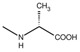	4.51
**6** (l-Val)	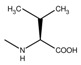	4.62
**7** (d-Val)	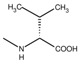	4.76
**8** (l-Ser)	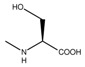	4.48
**9** (d-Ser)	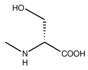	4.22
**14** (l-Met)	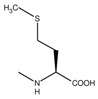	4.56
**15** (d-Met)	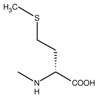	4.56
**16** (l-Phe)	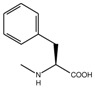	5.18
**17** (d-Phe)	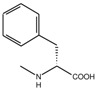	5.12
**18** (l-Tyr)	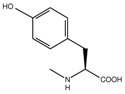	4.30
**19** (d-Tyr)	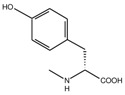	4.00
**20** (l-Trp)	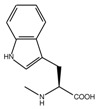	5.69
**21** (d-Trp)	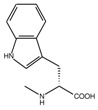	4.69

*^a^p*IC_50_ values are taken from [13].

**Table 2:** Total EphA2-inhibitor interaction energy ^*a*^ at the consecutive levels of theory.

Inhibitor	*p*IC${}_{50}$^*b*^	$E_{\mathrm{EL},\mathrm{MTP}}^{\left(10\right)}$	$E_{\mathrm{EL}}^{\left(10\right)}$	$E^{\left(10\right)}$	$E_{\mathrm{SCF}}$	$E_{\mathrm{MP}2}$	$E_{\mathrm{EL},\mathrm{MTP}}^{\left(10\right)}+E_{\mathrm{Das}}$
**20** (l-Trp)	5.69	−89.2	−101.3	−66.5	−83.5	−102.7	−118.0
**16** (l-Phe)	5.18	−90.7	−102.5	−65.6	−86.1	−100.5	−115.3
**17** (d-Phe)	5.12	−98.5	−111.4	−70.1	−92.6	−109.6	−127.0
**7** (d-Val)	4.76	−75.2	−83.3	−65.7	−77.4	−87.7	−91.3
**4** (l-Ala)	4.70	−97.1	−108.5	−73.7	−94.1	−103.5	−116.5
**21** (d-Trp)	4.69	−72.8	−82.3	−57.9	−70.9	−90.8	−99.4
**6** (l-Val)	4.62	−99.3	−110.0	−71.9	−94.4	−104.4	−120.4
**14** (l-Met)	4.56	−89.9	−101.1	−69.1	−87.7	−100.7	−112.3
**15** (d-Met)	4.56	−80.5	−89.5	−67.3	−80.6	−94.2	−101.5
**5** (d-Ala)	4.51	−75.1	−82.2	−66.7	−76.9	−85.6	−88.9
**8** (l-Ser)	4.48	−85.9	−96.6	−70.4	−86.2	−95.5	−103.7
**2** (Gly)	4.31	−64.6	−69.3	−56.2	−65.0	−72.5	−75.7
**18** (l-Tyr)	4.30	−65.9	−73.2	−55.3	−65.3	−79.4	−85.3
**9** (d-Ser)	4.22	−69.0	−74.7	−62.6	−71.4	−81.1	−83.2
**19** (d-Tyr)	4.00	−65.3	−74.1	−55.8	−66.5	−81.9	−85.7
R ^*c*^		−0.63	−0.65	−0.44	−0.55	−0.69	−0.72
$N_{pred}$ ^*d*^		75.0	76.9	65.4	69.2	75.0	77.9
*SE* ^*e*^		10.1	11.5	5.6	9.0	8.2	11.5

^*a*^ In units of $\;\mathrm{kcal}\cdot {\mathrm{mol}}^{-1}$; ^*b*^ $p{\mathrm{IC}}_{50}$ values are taken from [13]; ^*c*^ Correlation coefficient between the energy obtained at a given level of theory and the experimental inhibitory activity; ^*d*^ Percentage of successful predictions [%]; ^*e*^ Standard error of estimate, in units of $\;\mathrm{kcal}\cdot {\mathrm{mol}}^{-1}$.

**Table 3:** Solvation free energy ($\Delta G_{solv}$) of inhibitors of EphA2-ephrin A1 interaction with its electrostatic, $\Delta G_{solv,el}$, and non-electrostatic, $\Delta G_{solv,non-el}$, contributions ^*a*^.

Inhibitor	*p*IC${}_{50}$^*b*^	$\Delta G_{\mathrm{solv}}$	$\Delta G_{\mathrm{solv},\mathrm{el}}$	$\Delta G_{\mathrm{solv},\mathrm{non}-\mathrm{el}}$
**20** (l-Trp)	5.69	−73.6	−81.2	7.6
**16** (l-Phe)	5.18	−66.4	−73.5	7.2
**17** (d-Phe)	5.12	−67.9	−75.3	7.4
**7** (d-Val)	4.76	−63.2	−70.0	6.8
**4** (l-Ala)	4.70	−70.9	−77.0	6.0
**21** (d-Trp)	4.69	−67.5	−75.2	7.7
**6** (l-Val)	4.62	−68.6	−75.5	7.0
**14** (l-Met)	4.56	−69.0	−75.9	6.9
**15** (d-Met)	4.56	−66.3	−73.5	7.2
**5** (d-Ala)	4.51	−67.2	−73.4	6.2
**8** (l-Ser)	4.48	−66.2	−72.0	5.8
**2** (Gly)	4.31	−62.8	−68.1	5.3
**18** (l-Tyr)	4.30	−71.7	−78.9	7.2
**9** (d-Ser)	4.22	−64.2	−70.2	6.0
**19** (d-Tyr)	4.00	−67.2	−74.9	7.7
R ^*c*^		−0.43	−0.46	0.37

^*a*^ In units of $\;\mathrm{kcal}\cdot {\mathrm{mol}}^{-1}$; ^*b*^ $p{\mathrm{IC}}_{50}$ values are taken from [13]; ^*c*^ Correlation coefficient between the solvation free energy and the experimental inhibitory activity.

**Table 4:** Performance of $E_{MP2}$ and $E_{EL,MTP}^{\left(10\right)}+E_{Das}$ models and differences in ligand solvation free energy for EphA2-ephrin A1, menin-MLL [35], FAAH [33], and *Tb*PTR1 [34] inhibitors.

	EphA2-Ephrin A1	Menin-MLL	FAAH	*Tb*PTR1
$R_{MP2}$ ^*a*^	−0.69	−0.55	−0.83	−0.89
$R_{E_{EL,MTP}^{\left(10\right)}+E_{Das}}$ ^*b*^	−0.72	−0.87	−0.67	−0.96
SD ^*c*^	3.0	2.5	1.5	1.1

^*a*^ Correlation coefficient between the energy obtained at MP2 level of theory and the experimental inhibitory activity; ^*b*^ Correlation coefficient between the energy obtained with $E_{EL,MTP}^{\left(10\right)}+E_{Das}$ model and the experimental inhibitory activity; ^*c*^ $\Delta G_{solv}$ standard deviation within a given set of inhibitors. In units of $\;\mathrm{kcal}\cdot {\mathrm{mol}}^{-1}$.

**Table 5:** Performance of empirical scoring for FAAH, menin-MLL and EphA2-ephrin A1 systems. The results obtained for nonempirical $E_{EL,MTP}^{\left(10\right)}+E_{Das}$ model are provided for comparison.

Scoring Function	FAAH ^*a*^		menin-MLL ^*b*^		EphA2-ephrin A1 ^*c*^	
	R ^*d*^	$N_{\mathrm{pred}}$ ^*e*^	R	$N_{\mathrm{pred}}$	R	$N_{\mathrm{pred}}$
LigScore1	+0.25	44.6	−0.81	75.2	−0.80	79.6
Jain	−0.48	71.4	−0.80	77.8	−0.77	83.3
PLP2	−0.51	65.8	−0.79	80.4	−0.40	72.2
Ludi1	−0.62	73.2	−0.40	58.8	−0.71	75.9
PMF	−0.72	77.1	+0.24	41.2	−0.27	66.7
$E_{EL,MTP}^{\left(10\right)}+E_{Das}$	−0.67	74.9	−0.87	81.1	−0.79	79.6

^*a*^ The results are taken from [33]; ^*b*^ The results are taken from [35]; ^*c*^ The results refer to the reduced set of EphA2 inhibitors; ^*d*^ Correlation coefficient between the score obtained with a given empirical function or $E_{EL,MTP}^{\left(10\right)}+E_{Das}$ energy and the experimental inhibitory activity; ^*e*^ Percentage of successful predictions [%].

## Supplementary Materials

Supplementary materials are available online.
