# Supplementary material for: Theoretical Model of EphA2-Ephrin A1 Inhibition
Source: Molecules. 2018 Jul 11;23(7):1688. doi: 10.3390/molecules23071688 (PMC6099714; doi:10.3390/molecules23071688)
Supplement: Supplementary file 1 [file molecules-23-01688-s001.pdf]

Supplementary Materials: Theoretical Model of EphA2 – Ephrin A1 Inhibition

Wiktoria Jedwabny [1], Alessio Lodola [2] and Edyta Dyguda-Kazimierowicz [1]*

Table S1. Interaction energy values (in kcal · mol^{-1}) at the consecutive levels of theory for each amino acid residue-inhibitor pair.

Inhibitor	Residue	$E^{(10)}_{EL,MTP}$	$E^{(10)}_{EL}$	$E^{(10)}$	E_{SCF}	E_{MP2}	$E^{(10)}_{EL,MTP} + E_{Das}$
**20 (L-Trp)**	Cys70-Cys188	4.7	0.7	12.1	9.6	0.6	-5.0
	Phe108	-3.8	-5.7	0.4	-1.3	-6.0	-9.5
	Arg103	-88.2	-93.2	-79.4	-91.9	-93.4	-96.7
	Val72-Met73	-1.8	-3.2	0.4	-0.2	-3.9	-6.7
**16 (L-Phe)**	Cys70-Cys188	4.4	1.0	11.5	9.2	1.3	-4.3
	Phe108	-3.1	-3.9	-2.1	-3.3	-6.0	-6.6
	Arg103	-89.9	-98.0	-73.4	-90.4	-93.4	-101.9
	Val72-Met73	-1.6	-1.7	-1.7	-2.0	-2.3	-2.1
**17 (D-Phe)**	Cys70-Cys188	5.2	1.3	11.9	9.6	0.1	-5.2
	Phe108	-4.1	-5.5	-1.1	-2.9	-6.7	-9.0
	Arg103	-97.3	-104.7	-79.2	-97.6	-99.0	-107.8
	Val72-Met73	-2.0	-2.5	-1.6	-2.1	-4.1	-4.7
**7 (D-Val)**	Cys70-Cys188	4.1	0.2	11.4	8.9	0.8	-4.8
	Phe108	-2.7	-2.6	-2.5	-3.2	-3.8	-3.7
	Arg103	-74.7	-79.1	-72.9	-80.4	-82.6	-80.7
	Val72-Met73	-1.8	-1.8	-1.8	-2.0	-2.0	-1.9
**4 (L-Ala)**	Cys70-Cys188	4.5	1.3	11.5	8.9	1.8	-3.4
	Phe108	-3.1	-3.0	-2.8	-3.6	-3.9	-3.7
	Arg103	-97.8	-105.3	-80.7	-98.0	-99.7	-108.6
	Val72-Met73	-1.6	-1.6	-1.6	-1.8	-1.7	-1.7
**21 (D-Trp)**	Cys70-Cys188	4.6	-0.5	12.6	9.7	-0.4	-6.3
	Phe108	-2.9	-4.2	-0.9	-2.3	-6.9	-8.1
	Arg103	-72.0	-75.1	-70.3	-77.8	-80.1	-78.2
	Val72-Met73	-2.1	-2.5	0.6	-0.1	-3.3	-6.3
**6 (L-Val)**	Cys70-Cys188	4.3	1.3	9.8	7.9	1.0	-3.5
	Phe108	-2.5	-2.5	-2.4	-3.1	-3.7	-3.5
	Arg103	-99.4	-107.3	-77.7	-97.6	-100.0	-111.7
	Val72-Met73	-1.5	-1.6	-1.6	-1.8	-1.8	-1.7
**14 (L-Met)**	Cys70-Cys188	6.2	1.7	12.4	9.9	0.9	-3.9
	Phe108	-3.9	-4.3	-1.6	-3.0	-4.7	-6.2
	Arg103	-92.0	-97.1	-78.6	-93.1	-94.9	-101.4
	Val72-Met73	-1.4	-1.4	-1.3	-1.7	-2.1	-2.0
**15 (D-Met)**	Cys70-Cys188	4.9	1.6	11.6	9.1	0.8	-3.9
	Phe108	-4.4	-5.1	-1.5	-2.9	-5.6	-8.3
	Arg103	-79.9	-84.6	-76.2	-84.9	-87.5	-87.4
	Val72-Met73	-1.3	-1.4	-1.3	-1.5	-1.9	-2.0
**5 (D-Ala)**	Cys70-Cys188	3.9	0.3	10.6	8.4	0.9	-4.2

Table S1: continued from previous page

Inhibitor	Residue	$E^{(10)}_{EL,MTP}$	$E^{(10)}_{EL}$	$E^{(10)}$	E_{SCF}	E_{MP2}	$E^{(10)}_{EL,MTP} + E_{Das}$
**5 (D-Ala)**	Phe108	-2.5	-2.4	-2.4	-3.0	-3.3	-3.1
	Arg103	-74.9	-78.4	-73.2	-80.1	-81.5	-79.9
	Val72-Met73	-1.7	-1.7	-1.7	-1.9	-1.8	-1.8
**8 (L-Ser)**	Cys70-Cys188	5.2	2.4	11.5	9.3	2.1	-2.8
	Phe108	-3.4	-3.3	-3.0	-4.1	-4.5	-4.4
	Arg103	-88.3	-93.8	-77.0	-89.5	-91.0	-97.1
	Val72-Met73	-1.8	-1.8	-1.8	-2.1	-2.1	-2.0
**2 (Gly)**	Cys70-Cys188	5.2	2.3	10.7	8.4	2.5	-0.5
	Phe108	-2.4	-2.4	-2.4	-3.0	-3.0	-2.7
	Arg103	-65.1	-67.4	-62.7	-68.0	-70.2	-70.2
	Val72-Met73	-1.8	-1.8	-1.8	-2.0	-1.8	-1.9
**18 (L-Tyr)**	Cys70-Cys188	2.0	-1.3	10.2	7.7	-0.5	-7.0
	Phe108	-3.3	-4.3	-1.9	-3.0	-5.9	-7.0
	Arg103	-63.8	-66.0	-62.0	-67.9	-70.9	-70.1
	Val72-Met73	-1.6	-1.6	-1.6	-1.8	-2.1	-2.1
**9 (D-Ser)**	Cys70-Cys188	5.4	2.0	11.4	9.3	1.3	-3.5
	Phe108	-3.3	-3.2	-3.1	-4.0	-4.5	-4.2
	Arg103	-69.7	-71.5	-68.9	-74.2	-75.8	-74.1
	Val72-Met73	-1.9	-1.9	-1.9	-2.2	-2.1	-2.1
**19 (D-Tyr)**	Cys70-Cys188	4.2	0.4	11.1	8.6	0.4	-4.3
	Phe108	-3.5	-4.6	-2.1	-3.4	-6.7	-7.8
	Arg103	-65.7	-68.2	-63.1	-69.6	-73.3	-72.6
	Val72-Met73	-1.7	-1.6	-1.6	-1.8	-2.3	-2.4

Table S2. Free energy of solvation (ΔG_{solv}) of FAAH inhibitors with its electrostatic ($\Delta G_{solv,el}$) and non-electrostatic ($\Delta G_{solv,non-el}$) contributions.[a]

Inhibitor[b]	ΔG_{solv}	$\Delta G_{solv,el}$	$\Delta G_{solv,non-el}$
**4m**	-5.8	-11.5	5.8
**4g**	-6.5	-10.5	4.0
**4b**	-5.5	-10.5	5.0
**4d**	-5.5	-10.1	4.6
**4r**	-6.8	-12.9	6.1
**4v**	-7.7	-15.2	7.5
**4s**	-6.5	-13.1	6.6
**4l**	-6.6	-11.7	5.1
**4t**	-12.0	-16.0	4.0
**4i**	-5.5	-10.1	4.6
**4k**	-4.9	-9.2	4.3
**4a**	-5.9	-9.7	3.8
**2**	-5.5	-10.0	4.5
**4u**	-6.0	-13.2	7.2
**4p**	-6.6	-11.9	5.4
**4h**	-5.8	-10.0	4.2
**4n**	-6.5	-11.8	5.4
**4c**	-5.1	-9.7	4.6
**4e**	-4.9	-10.2	5.3
**4q**	-7.1	-12.8	5.7
**4o**	-6.9	-12.1	5.2
**4f**	-4.5	-10.4	5.9

[a] In units of $\mathrm{kcal \cdot mol^{-1}}$.
[b] Inhibitors presented in ref. 1.

Table S3. Free energy of solvation (ΔG_{solv}) of *Tb*PTR1 inhibitors with its electrostatic ($\Delta G_{solv,el}$) and non-electrostatic ($\Delta G_{solv,non-el}$) contributions.[a]

Inhibitor[b]	ΔG_{solv}	$\Delta G_{solv,el}$	$\Delta G_{solv,non-el}$
**fr-C12**	-2.1	-6.5	4.4
**fr-C11**	-2.5	-6.8	4.3
**fr-C31**	-4.3	-9.4	5.1
**fr-C9**	-1.2	-4.0	2.8
**fr-C10**	-1.3	-4.1	2.8
**fr-C33**	-2.5	-6.5	4.0

[a] In units of $\mathrm{kcal \cdot mol^{-1}}$.
[b] Inhibitors presented in ref. 2.

Table S4. Free energy of solvation (ΔG_{solv}) of inhibitors menin-MLL interaction with its electrostatic ($\Delta G_{solv,el}$) and non-electrostatic ($\Delta G_{solv,non-el}$) contributions.[a]

Inhibitor[b]	ΔG_{solv}	$\Delta G_{solv,el}$	$\Delta G_{solv,non-el}$
**MI-2-2**	-16.4	-17.7	1.3
**MI-859**	-15.4	-16.2	0.8
**MI-319**	-16.1	-19.2	3.1
**MI-2-3**	-14.9	-18.6	3.7
**MI-836**	-16.0	-16.3	0.3
**MI-2**	-14.6	-15.0	0.4
**MI-273**	-10.4	-12.7	2.3
**MI-20**	-14.4	-14.7	0.2
**MI-2-4**	-13.8	-15.3	1.6
**MI-326**	-16.7	-18.9	2.2
**MI-19**	-14.7	-14.5	-0.3
**MI-333**	-16.5	-19.2	2.7
**MI-12**	-8.7	-12.0	3.4
**MI-16**	-12.6	-14.0	1.4
**MI-4**	-8.5	-11.8	3.3
**MI-10**	-13.1	-17.4	4.3
**MI-11**	-12.6	-14.3	1.7
**MI-6**	-15.3	-16.1	0.9

[a] In units of $\mathrm{kcal \cdot mol^{-1}}$.
[b] Inhibitors presented in ref. 3.

Table S5. The performance of the nonempirical models of inhibitory activity within the reduced and full EphA2 inhibitor sets.

Model	Descriptor	$E^{(10)}_{EL,MTP}$	$E^{(10)}_{EL}$	$E^{(10)}$	E_{SCF}	E_{MP2}	$E^{(10)}_{EL,MTP} + E_{Das}$
Full	R	-0.63	-0.65	-0.44	-0.55	-0.69	-0.72
	N_{pred}	75.0	76.9	65.4	69.2	75.0	77.9
Reduced	R	-0.71	-0.73	-0.45	-0.64	-0.78	-0.79
	N_{pred}	77.8	81.5	64.8	70.4	77.8	79.6

[a] Correlation coefficient between the energy obtained at a given level of theory and the experimental inhibitory activity taken from Ref. 4.
[b] Percentage of successful predictions [%].

Table S6. The performance of empirical scoring functions within the reduced and full EphA2 inhibitor sets. Inhibitors denoted in gray were removed to constitute the reduced model.

Inh.	pIC$_{50}$[a]	SASA[b]	$S_{L/L}$[b]	$S_{H/H}$[b]	GoldScore[c]	ChemScore[c]	ASP[c]	ChemPLP[d]	Vina[d]	LigScore1[e]	PLP2[c]	Jain[c]	PMF04[c]	PMF[c]	Ludi1[c]	Ludi3[c]	Glide SP[d]
20	5.69	479.1	53.3	26.9	26.9	10.7	16.1	-60.1	-3.1	2.5	32.1	0.8	33.3	67.0	164	297	-2.3
**16**	5.18	431.3	40.8	23.9	24.8	10.4	13.0	-61.4	-2.5	2.3	28.4	0.4	33.3	71.4	171	264	-2.4
**17**	5.12	450.0	42.5	28.9	25.7	13.1	16.7	-54.2	-2.7	2.6	27.4	1.5	34.3	64.8	243	299	-2.7
7	4.76	377.1	17.6	21.3	16.6	4.8	8.4	-49.2	-1.7	1.5	19.2	-2.9	21.9	51.6	-4	67	-1.7
**4**	4.70	326.0	9.0	40.1	17.9	5.9	7.9	-47.7	-1.7	2.1	24.2	0.5	19.9	45.4	215	249	-2.3
**21**	4.69	486.7	37.0	26.1	29.7	12.5	18.1	-62.7	-3.6	1.8	31.5	-1.8	33.3	78.2	88	254	-1.8
**6**	4.62	369.5	22.6	34.7	14.3	7.5	10.1	-51.9	-1.8	2.4	24.6	0.0	29.7	62.9	196	221	-2.8
**14**	4.56	413.3	18.0	33.5	21.8	6.9	9.8	-54.4	-2.0	2.3	27.1	-0.2	27.2	53.7	104	166	-1.7
**15**	4.56	415.4	22.9	31.0	22.2	5.3	9.4	-51.2	-1.8	2.0	22.6	-2.0	27.4	53.4	-16	81	-1.1
**5**	4.51	331.1	6.3	29.7	15.1	4.4	8.1	-46.2	-1.8	1.3	16.2	-2.5	14.9	36.5	29	89	-1.4
**8**	4.48	342.7	12.2	38.4	13.8	2.1	9.5	-51.8	-1.6	2.0	19.3	-0.7	23.2	52.5	100	162	-1.5
2	4.31	313.7	7.3	30.4	13.1	2.2	4.8	-45.8	-1.2	0.3	12.8	-1.5	13.3	35.6	5	91	-0.8
18	4.30	456.3	32.2	18.4	18.1	10.6	16.2	-55.9	-2.6	0.8	26.5	-1.9	23.6	59.5	5	149	-1.4
**9**	4.22	345.8	7.6	33.0	13.4	1.1	8.1	-53.6	-1.7	1.1	14.5	-2.0	18.9	38.4	-13	79	-1.1
**19**	4.00	463.2	42.2	20.1	21.3	6.9	14.4	-55.8	-2.6	1.0	28.2	-2.5	32.1	74.0	32	137	-1.3
$R_{(f)}$[f]		-0.36	-0.55	0.01	-0.59	-0.57	-0.38	-0.41	-0.44	-0.71	-0.51	-0.68	-0.50	-0.36	-0.63	-0.71	-0.69
$N_{pred(f)}$[f]		59.6	66.4	49.0	72.1	72.1	60.6	57.7	61.5	77.9	69.2	75.0	69.2	64.4	70.2	75.0	77.9
$R_{(r)}$[g]		-0.20	-0.36	-0.01	-0.48	-0.67	-0.33	-0.27	-0.29	-0.80	-0.40	-0.77	-0.40	-0.27	-0.71	-0.75	-0.73
$N_{pred(r)}$[g]		55.6	66.7	48.2	74.1	81.5	61.1	55.6	61.1	79.6	72.2	83.3	72.2	66.7	75.9	85.2	81.5

[a] pIC$_{50}$ values are taken from ref. 4.
[b] In units of Å^2.
[c] In arbitrary units.
[d] In $\mathrm{kcal \cdot mol^{-1}}$.
[e] In pK$_i$
[f] Correlation coefficient between the energy obtained at a given level of theory and the experimental inhibitory activity or the percentage of successful predictions obtained for the full set of inhibitors.
[g] Correlation coefficient between the energy obtained at a given level of theory and the experimental inhibitory activity or the percentage of successful predictions obtained for the reduced set of 11 inhibitors indicated by names given in black bold font.

Details regarding scoring with empirical functions

- **SASA**: The probing sphere of the molecule surface was set to 1.4Å radius.
- **Platinum**: Molecular Hydrophobicity Potential (MHP) parameters, i.e., MHP distance function, atomic hydrophobicity parametrization and the offset of the MHP scale were chosen with default settings.
- **Discovery Studio 2017**: Binding site radius was equal to 10Å, and the sphere was centered on the ligand, yielding the following points of origin (in units of Å):

  - L-Trp: -32.68 -5.19 23.73
  - L-Phe: -32.92 -5.64 23.83
  - D-Phe: -32.65 -4.99 23.50
  - D-Val: -34.17 -6.00 23.11
  - L-Ala: -35.21 -6.16 23.30
  - D-Trp: -33.04 -5.11 23.68
  - L-Val: -33.99 -6.36 23.54
  - L-Met: -33.95 -5.74 23.13
  - D-Met: -33.97 -5.71 23.49
  - D-Ala: -35.40 -6.02 23.18
  - L-Ser: -34.80 -6.16 23.40
  - Gly: -36.01 -6.51 23.47
  - L-Tyr: -33.50 -5.81 23.78
  - D-Ser: -34.89 -5.88 23.18
  - D-Tyr: -33.41 -5.72 23.92

  All scoring functions' parameters were set to default values, i.e.: Momany-Rone was selected as a Ligand Partial Charge Method; Dreiding LigScore Forcefield was chosen with Energy Grid Extension equal to 5.0; PMF Carbon-Carbon and Other Interactions Cutoff were set to 12.0Å; PMF04 Carbon-Carbon and Other Interactions Cutoff were set to 6.0 and 9.0Å, respectively.
- **Gold 4.0**: Binding site radius was equal to 10Å, and the sphere was centered on the alpha carbon of Arg103; All receptor residues were set rigid; Gold default parameter files were used for each scoring function. These define external H-bond, external vdw, internal vdw, and internal torsion for GoldScore; hydrogen bond, metal, lipophilic, clash, and torsion scaled terms for ChemScore; hydrogen bond, clash, and torsion scaled terms for ASP, as well as the ASP_GRID_SPACING parameter (equal to 0.3Å), controlling the density of the precalculated grid used for evaluation of the atom-atom potentials.
- **AutoDock Vina**: Grid was set with 0.375Å spacing, with 60 points in each direction, yielding 22.5Å cubic grid centered on the ligand (coordinates of the grid center were chosen to match the corresponding values from Discovery Studio 2017); All receptor residues were set rigid; Weighting values of gauss 1, gauss2, repulsion, hydrophobic, and hydrogen parameters were set default during scoring.
- **Plants**: Program version 1.2 was used. Search speed was set to 1; Binding site radius was equal to 10Å, and the sphere was centered on the ligand (coordinates of the sphere origin were chosen to match the corresponding values from Discovery Studio 2017).
- **Glide**: 15Å cubic grid was centered on the ligand; All hydroxyl and thiol groups within the grid were allowed to rotate, and no constraints were applied; During scoring, GlideScore version SP5.0 parameters were used; Van der Waals radius scaling and partial charge cut-off were set default (0.80 scaling factor and 0.15Å, respectively), Epik state penalties were allowed, and OPLS3 force field was applied.

Bibliography

1. Giedroyć-Piasecka, W.; Dyguda-Kazimierowicz, E.; Beker, W.; Mor, M.; Lodola, A.; Sokalski, W.A. Physical Nature of Fatty Acid Amide Hydrolase Interactions with Its Inhibitors: Testing a Simple Nonempirical Scoring Model. *Journal of Physical Chemistry B* **2014**, *118*, 14727–14736.

2. Jedwabny, W.; Panecka-Hofman, J.; Dyguda-Kazimierowicz, E.; Wade, R.C.; Sokalski, W.A. Application of a simple quantum chemical approach to ligand fragment scoring for Trypanosoma brucei pteridine reductase 1 inhibition. *Journal of Computer-Aided Molecular Design* **2017**, *31*, 715–728.
3. Jedwabny, W.; Kłossowski, S.; Purohit, T.; Cierpicki, T.; Grembecka, J.; Dyguda-Kazimierowicz, E. Theoretical models of inhibitory activity for inhibitors of protein-protein interactions: targeting menin-mixed lineage leukemia with small molecules. *Med. Chem. Commun* **2017**, *8*, 2216–2227.
4. Incerti, M.; Tognolini, M.; Russo, S.; Pala, D.; Giorgio, C.; Hassan-Mohamed, I.; Noberini, R.; Pasquale, E.B.; Vicini, P.; Piersanti, S.; Rivara, S.; Barocelli, E.; Mor, M.; Lodola, A. Amino Acid Conjugates of Lithocholic Acid As Antagonists of the EphA2 Receptor. *Journal of Medicinal Chemistry* **2013**, *56*, 2936–2947.
